# Novel Bayesian Inference-Based Approach for the Uncertainty Characterization of Zhang’s Camera Calibration Method

**DOI:** 10.3390/s23187903

**Published:** 2023-09-15

**Authors:** Ramón Gutiérrez-Moizant, María Jesús L. Boada, María Ramírez-Berasategui, Abdulla Al-Kaff

**Affiliations:** 1 Mechanical Engineering Department, Universidad Carlos III de Madrid, Avda. de la Universidad 30, 28911 Leganés, Spain; mjboada@ing.uc3m.es (M.J.L.B.); mrami@ing.uc3m.es (M.R.-B.); 2 Systems Engineering and Automation, Universidad Carlos III de Madrid, Avda. de la Universidad 30, 28911 Leganés, Spain; akaff@ing.uc3m.es

**Keywords:** camera calibration, computer vision, uncertainty quantification, Bayesian inversion

## Abstract

Camera calibration is necessary for many machine vision applications. The calibration methods are based on linear or non-linear optimization techniques that aim to find the best estimate of the camera parameters. One of the most commonly used methods in computer vision for the calibration of intrinsic camera parameters and lens distortion (interior orientation) is Zhang’s method. Additionally, the uncertainty of the camera parameters is normally estimated by assuming that their variability can be explained by the images of the different poses of a checkerboard. However, the degree of reliability for both the best parameter values and their associated uncertainties has not yet been verified. Inaccurate estimates of intrinsic and extrinsic parameters during camera calibration may introduce additional biases in post-processing. This is why we propose a novel Bayesian inference-based approach that has allowed us to evaluate the degree of certainty of Zhang’s camera calibration procedure. For this purpose, the a prioriprobability was assumed to be the one estimated by Zhang, and the intrinsic parameters were recalibrated by Bayesian inversion. The uncertainty of the intrinsic parameters was found to differ from the ones estimated with Zhang’s method. However, the major source of inaccuracy is caused by the procedure for calculating the extrinsic parameters. The procedure used in the novel Bayesian inference-based approach significantly improves the reliability of the predictions of the image points, as it optimizes the extrinsic parameters.

## 1. Introduction

Accurate knowledge of image projection parameters is essential for computer vision and photogrammetry applications. These techniques are the science of extracting information about the physical world from image data. Photogrammetry mainly involves the precise measurement of three-dimensional objects and terrain features from two-dimensional photos [1]. Topographical mapping, architecture, engineering, manufacturing, quality control, police investigation, defense, agriculture, geology, film and games industries are some of the areas where photogrammetry is used [2].

On the other hand, computer vision focuses on developing systems that can capture, process and analyze visual data to understand the world around them for scientific and technical exploration [3]. Computer vision is used in a wide range of industrial and scientific applications, such as robot and autonomous vehicles, control systems, traffic speed, automatic inspection in manufacturing applications, medical image processing or computer–human interaction [4].

Usually, the information from the camera manufacturer is not accurate enough to be of use in either photogrammetry or computer vision applications [5]. To accurately reconstruct the camera’s mapping from the 3D world to the 2D image, it is necessary to determine the relationship between the three-dimensional coordinates in the real world and the pixel of the captured image. To determine this, the calibration of the camera is required. Camera calibration is the process of characterizing the internal properties or interior orientation of the camera (intrinsic parameters and focal lens distortion) and determining the position and orientation of the camera with respect to the captured scene (extrinsic parameters or exterior orientation) [1]. Once the parameters of the camera are known, the theoretical camera model can be used to extract metric information from 2D images [6].

The calibration of the camera has always been an important issue in both photogrammetry and computer vision applications. Although, from the point of view of photogrammetry, the problem of camera calibration is considered to be solved [7], the calibration methods used in computer vision have become very popular due to their ease of application [8]. However, Luhmann et al. [7] warns that little attention is paid to the accuracy of the calibration methods used in computer vision. For instance, the focus of computer vision researchers has centered on the development of user-friendly, automated calibration procedures based on linear methods with simplified image models.

In the past, cameras were calibrated separately. Currently, object modeling using a set of images from different camera perspectives is a very extended practice [9,10]. Moreover, in situ calibration can also be performed by applying deep learning methods [11,12], based on vanishing points [13]. However, the vanishing point approach assumes that the camera is undistorted [12], and the internal parameters are considered fixed, i.e., without uncertainty [11].

Separate calibration or pre-calibration is still required for single camera applications such as special survey cameras or aerial cameras [7].

There are several methods used for single camera calibration in the computer vision community [6,9,14,15,16]. The aim of these methods is to find the best estimate of the camera parameters that minimizes the difference between the image points and the estimated points. However, according to [17], the ’best’ answer is ambiguous because of the uncertainty involved. Furthermore, this is because, according to metrology, a measurement is incomplete if it is not presented with its corresponding uncertainty [18]. In this sense, one of the challenges in modern metrology is to provide the mathematical tools to quantify the uncertainties of inverse and non- linear problems in scientific modeling [19]. In fact, this is a research hotspot where the solutions presented to date combine different mathematical algorithms [20]. Among them, the Bayesian method has proved its worth in the inverse uncertainty inference [21,22,23]. According to King et al. [24], the Bayesian approach represents reality more accurately than the uncertainties obtained using the standard frequentist approach, where it is commonly assumed that the probability density function (PDF) of the uncertainties are Gaussian.

The most widely used method for camera calibration is the one proposed by Zhang [6], due to its ease of application, efficiency and robustness [25,26]. In this method, 2 to 5 images of different poses of a planar checkerboard are required to determine the interior orientation of the camera [27]. The camera and planar pattern can move freely and the movement does not have to be known. However, it was observed that the values of the interior orientation depend on the number of images captured [27], so it is recommended to use a large number of images for a better adjustment of these parameters. Additionally, the previous studies [28,29,30] have shown that the accuracy of the calibration results is highly dependent on the poses used in the acquired images. According to [29], inexperienced users often do not take calibration images that enhance calibration accuracy. Therefore, refs [28,29,30] propose interactive methods that help to successfully perform camera calibration. In these methods, all target poses that lead to a reduction in the interior camera orientation uncertainty are preferred and the rest are ignored.

It is well known that the ground truth of the calibration parameters is unfortunately unknown in reality. However, it is usually assumed that it can be approximated from a large number of images or calibration experiments [31]. Typically, the accuracy of calibration results is measured by examining re-projection errors. High values may indicate large systematic errors or inaccurate calibration. Nevertheless, they cannot give a complete information about the quality of the calibration [32]. Some camera calibration software packages, such as those implemented in Matlab [33] or OpenCV [34], also calculate the expanded uncertainty of both interior and exterior camera parameters based in Zhang’s method [6] for a 95% confidence level, assuming they follow a normal probability distribution. These uncertainties are calculated from the covariance matrix, which is computed considering the Jacobian matrix of the calibration residuals and the mean square error [35].

Therefore, to determine the uncertainty of the camera parameters using Zhang’s method [6], it is assumed that: (a) the uncertainties can be captured by the variability of the points used in the non-linear regression model, and (b) the uncertainties depend on the images considered in the calibration.

The results of camera calibration using the software mentioned above should be interpreted with caution for the following reasons:

- The method of estimating parameter uncertainties leads to the assumption that the model is linear, when, in fact, it is highly non-linear.
- Although there are studies dedicated to the analysis of the uncertainty of camera parameters [31,32,36,37], none of them have verified the hypothesis of normality of the calibrated parameters.
- The accuracy of the parameters with these software has not been verified with a suitable alternative method.

Therefore, the aim of this research work is to address the above underlined weaknesses commonly accepted by the computer vision community. To this end, a novel Bayesian inference-based method is proposed to validate the intrinsic parameters and lens distortion coefficients (interior orientation) obtained with the calibration process of Zhang [6]. Moreover, the new method proposed allows us to update the information of the intrinsic parameters and lens distortion coefficients with the different sets of images considered. It also improves the accuracy and reliability of the calibration process by minimizing re-projection errors. This article is organized as follows. Section 2 provides a brief description of Zhang´s camera calibration procedure. Section 3 describes the proposed Bayesian inversion calibration of the interior camera orientation parameters. In Section 4 an uncertainty propagation of the interior camera orientation parameters through the pinhole model is developed to evaluate the accuracy of the estimated image points. Finally, Section 5 summarizes the main results and key remarks.

## 2. Materials and Methods

### 2.1. Camera Calibration

The pinhole model is the most extended camera model [38], which relates world points to image points. In the original pinhole model [39], the camera is modeled by a set of intrinsic parameters (focal length, principal point, axis skew), and its poses and orientation are expressed by the extrinsic or exterior parameters (rotation matrix and translation vector). However, subsequent works have improved the model by including lens distortion in the internal camera parameters (interior orientation in photogrammetry) [39,40,41,42]. The intrinsic parameters are responsible for transforming the 3D points of the camera reference system, or camera coordinates, into 2D points of the displayed image. On the other hand, the extrinsic parameters provide information about the position of the camera with respect to the captured scene and transform the coordinates of the world into the camera reference system. The relationship between image points ${\left[u,v\right]}^{T}$ with respect to real world point coordinates ${\left[X,Y,Z\right]}^{T}$ can be expressed as follows:

(1) $$s\left[\begin{array}{c} u\\ v\\ 1 \end{array}\right]=A\left[\begin{array}{cc} R & t \end{array}\right]\left[\begin{array}{c} X\\ Y\\ Z\\ 1 \end{array}\right],$$

where *s* is a scaling factor, (R,t) are the extrinsic or exterior parameters composed by the rotation and translation matrices, respectively, and A is the intrinsic parameter matrix defined by:

(2) $$A=\left[\begin{array}{ccc} f_{x} & \gamma & c_{x}\\ 0 & f_{y} & c_{y}\\ 0 & 0 & 1 \end{array}\right],$$

$f_{x}$ and $f_{y}$ are image axis scale factors, $c_{x}$ and $c_{y}$ are principal point coordinates, and γ is the skewness of the *u* and *v* image axes.

Transforming 3D world coordinates to camera coordinates is expressed as follows:

(3) $$\left[\begin{array}{c} X_{c}\\ Y_{c}\\ Z_{c} \end{array}\right]=R\left[\begin{array}{c} X\\ Y\\ Z \end{array}\right]+t,$$

The next step is to perform the projection of the 3D camera points from the camera coordinates to the 2D pixel coordinates:

(4) $$s\left[\begin{array}{c} u\\ v\\ 1 \end{array}\right]=\left[\begin{array}{ccc} f_{x} & \gamma & c_{x}\\ 0 & f_{y} & c_{y}\\ 0 & 0 & 1 \end{array}\right]\left[\begin{array}{c} x^{\prime }\\ y^{\prime }\\ 1 \end{array}\right],$$

where $x^{\prime }=X_{c}/Z_{c}$ and $y^{\prime }=Y_{c}/Z_{c}$ are the camera’s homogeneous coordinates.

The pinhole model is an approximation of the camera projection expressing with a simple mathematical formulation the relationship between the object and image coordinates [16]. However, it is not valid for modeled real cameras. To accurately represent a real camera, lens distortion must be included in the pinhole model [39,40,41,42]. Brown [40] divided the distortion of a lens into radial distortion and tangential distortion. According to Brown [40], the radial distortion can be modeled by three parameters: $k_{1}$, $k_{2}$ and $k_{3}$. On the other hand, the tangential distortion is characterized with three coefficients: $p_{1}$, $p_{2}$ and $p_{3}$. In practice, only the first two terms of tangential distortion are considered, as the remaining terms are usually negligible [42]. Thus, for a more comprehensive camera model, the distorted points can be estimated by the following empirical equations [37]:

(5) $$\begin{array}{c} \;x^{\prime \prime }=x^{\prime }\left(1+k_{1}r^{2}+k_{2}r^{4}+k_{3}r^{6}\right)+2p_{1}x^{\prime }y^{\prime }+p_{2}\left(r^{2}+2{x^{\prime }}^{2}\right)\\ y^{\prime \prime }=y^{\prime }\left(1+k_{1}r^{2}+k_{2}r^{4}+k_{3}r^{6}\right)+p_{1}\left(r^{2}+2{y^{\prime }}^{2}\right)+2p_{2}x^{\prime }y^{\prime } \end{array},$$

with $r={x^{\prime }}^{2}+{y^{\prime }}^{2}$.

Using in Equation (4) the normalized distorted points of the image shown in Equation (5) instead of the ideal $x^{\prime }$ and $y^{\prime }$, the coordinates of the 3D projection point in the 2D image points are obtained:

(6) $$\begin{array}{cc} u & =f_{x}\cdot x^{\prime \prime }+{\gamma \cdot y^{\prime \prime }+c}_{x}\\ v & =f_{y}\cdot y^{\prime \prime }+c_{y} \end{array},$$

### 2.2. Plane-Based Camera Calibration

Zhang’s camera calibration method is one of the most commonly used. This calibration procedure is briefly described below [27]:

1. Take *n* images of the planar calibration pattern at different orientations by moving either the plane, the camera or both.
2. In each image, *m* feature points (corners) are detected. With these, the associated image homographies H are computed.
3. Using the homographies, the intrinsic parameters A are estimated by least- squares minimization.
4. The radial and tangential distortion coefficients are solved by the method of least squares.
5. With the interior orientation, the undistorted points and the world points, the extrinsic or exterior orientation parameters (R,t) are calculated.
6. Finally, all the estimated parameters values are refined by non-linear optimization (Levenberg–Marquardt algorithm), considering all the *m* corners of the *n* views.
  (7) $$\sum_{i=1}^{n}\sum_{j=1}^{m}{∥m_{i,j}-\overset{˘}{m}\left(A,k_{1},k_{2},k_{3},p_{1},p_{2},R_{i},t_{i},M_{j}\right)∥}^{2},$$
  where $\overset{˘}{m}\left(A,k_{1},k_{2},k_{3},p_{1},p_{2},R_{i},t_{i},M_{j}\right)$ is the estimated projection of the 2D image point $m_{i,j}$, corresponding to the detected target points $M_{j}={\left[X_{j},\;Y_{j},Z\right]}^{T}$.

**Figure 1:**
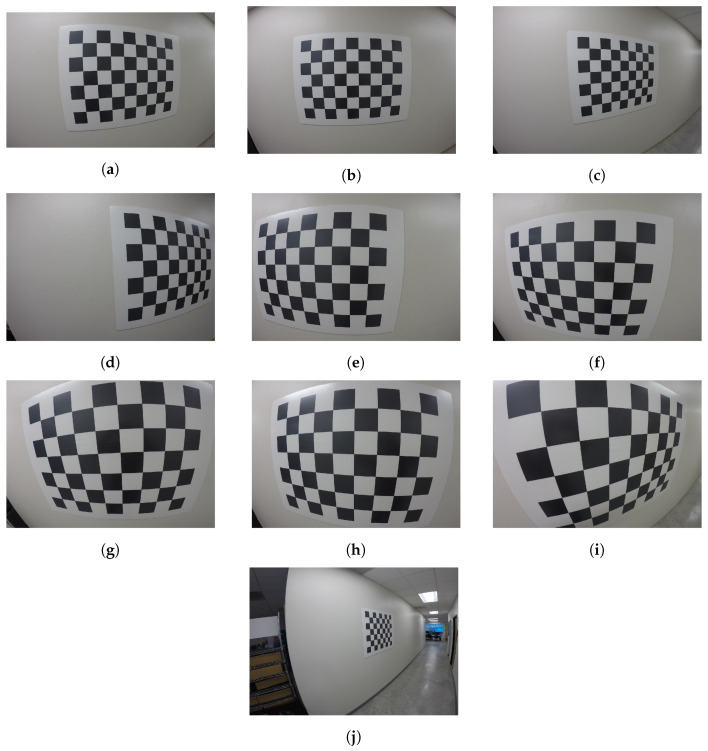
Multiple images of the planar checkerboard pattern in different poses for camera calibration [43].

In this paper, we have used the Matlab camera calibration toolbox [33] based on Zhang’s method. For the calibration, we have downloaded from the GitHub repository [43] 10 images of a 48-corner checkerboard, which were taken by a sports camera. As this method requires at least 2 views of the planar calibration target with different orientations, the 10 images were separated in pairs (5 sets). The calibration of the 5 sets was performed separately in sequence, i.e., set 1 = [Figure 1a,b], set 2 = [Figure 1c,d], ⋯, set 5 = [Figure 1i,j].

## 3. Bayesian Inference-Based Method for Uncertainty Characterization

An inverse model based on Bayesian inference was performed to validate the uncertainties of the interior camera orientation results of the Matlab toolbox. Moreover, the Bayesian method allows for the determination of the uncertainties of a mathematical model. It is based on a prior assumption (belief or knowledge) about the probability distributions of the uncertain parameters involved in the model [44]. It is, therefore, possible to speak of the establishment of a priori probability density functions (PDFs), based on the knowledge of the phenomenon being represented (in this case, the uncertainties of the interior camera orientation obtained with the Matlab toolbox) and of a posteriori or updated PDFs. In Bayesian inference, the prior experimental observations determine the subsequent observations and, thus, the final distribution [45].

In the proposed Bayesian method of camera calibration, only the interior camera orientations are evaluated, since the extrinsic parameters or exterior camera orientations can be obtained from them [6]. Furthermore, since the complete camera model is non-linear, due to lens distortion, the PDFs of the extrinsic parameters obtained using the Matlab toolbox cannot be employed for either Bayesian inversion or uncertainty propagation, as they are not independent. They depend on the values of the intrinsic camera parameters and lens distortion [6].

Bayesian inference is based on the rules of Bayes’ theorem, which, for our analysis, reads as follows:

(8) $$\pi \left(X\left|m\right.\right)=\frac{L\left(m\left|X\right.\right)\pi \left(X\right)}{Z},$$

where $\pi \left(X\left|m\right.\right)$ is the posterior distribution of the parameters:

$$\begin{array}{c} X=\left(A,k_{1},k_{2},k_{3},p_{1},p_{2}\right) \end{array}$$

which is conditional on the actual known values, in this case, the checkerboard 2D image points or reference points $m=[u_{i,j},v_{i,j}]$. This means that the probability distributions of the X parameters are updated using the experimental data m. The interior camera orientation X is composed by the intrinsic camera parameters A and the radial and tangential distortion coefficients $\left(k_{1},k_{2},k_{3},p_{1},p_{2}\right)$. $L\left(m\left|X\right.\right)$ is the likelihood function between the theoretical or estimated image points M(X), i.e., u,v from Equation (6), and the reference points m. π(X) is the a priori probability distribution, representing the belief or knowledge of the uncertain parameters, in our case, the interior camera orientation uncertainties (Gaussian PDFs), obtained with the Matlab camera calibration software. Moreover, *Z* is a normalized constant representing the model evidence or marginal likelihood.

Any mathematical or computational model is an incomplete representation of reality due to its simplifications. Therefore, there is a discrepancy term ϵ associated with the model M(X) that justifies its mismatch with the observed data m [46]. The relationship between the actual measurements and the image point estimated with the model is

(9) m=M(X)+ϵ

In general, it is assumed that the discrepancy will follow a Gaussian distribution $\epsilon \;N\left(0,\sigma \right)$. This discrepancy term represents the effects of both measurement error and model inaccuracy [47]. In many practical situations, it is difficult to perfectly know σ. According to [46], if the residual variance is unknown, it can be estimated from the residual between the estimated and experimental data. Therefore, the residual variance can be defined as

(10) $$\sigma^{2}=\frac{1}{n\times m}\sum_{i=1}^{n}\sum_{j=1}^{m}{\epsilon_{i,j}}^{2}=\frac{1}{n\times m}\sum_{i=1}^{n}\sum_{j=1}^{m}{\left(m_{i,j}-M\left(X\right)\right)}^{2},$$

where the variance $\sigma^{2}$ is now treated as an independent uncertain parameter with a priori distribution $\pi (\sigma^{2})$ [47]. Combining the a priori distribution of the variance $\pi (\sigma^{2})$ and the a priori distribution of the uncertain parameters of the model π(X), the following joint prior distribution is obtained [47]:

(11) $$\pi \left(X,\sigma^{2}\right)=\pi \left(X\right)\pi \left(\sigma^{2}\right),$$

the joint prior distribution $\pi (X,\sigma^{2})$ allows $\pi (\sigma^{2})$ to be included in the inference process. Thus, its PDF is also updated in conjunction with π(X). Therefore, instead of using Equation (8), the following posterior distribution should be used:

(12) $$\pi \left(X,\sigma^{2}\left|m\right.\right)=\frac{L(m\left|X,\sigma^{2}\right)\pi \left(X,\sigma^{2}\right)}{Z}=\frac{L(m\left|X,\sigma^{2}\right)\pi \left(X\right)\pi \left(\sigma^{2}\right)}{Z}$$

According to [47], the traditional likelihood functions for the case of the unknown residual variance problem is given by

(13) $$L\left(m\left|X,\sigma^{2}\right.\right)=\prod_{i=1}^{N}\frac{1}{\sqrt{2\pi \sigma^{2}}}\times exp\left(-\frac{1}{2\sigma^{2}}{\left(m_{i}-M\left(X\right)\right)}^{T}\left(m_{i}-M\left(X\right)\right)\right),$$

where *N* is the number of experimental data points, i.e., the total number of image points or references points for all the poses of the checkerboard. The likelihood function quantifies the degree of accuracy of the predicted image points M(X) with respect to the checkerboard corners $m_{i}$ for the X values considered. A higher degree of accuracy implies a greater influence of the sample X on the a posteriori distribution (Equation (8)), i.e., the likelihood functions are used to update the last distribution.

For each set of random samples $\left\{X^{\left(1\right)},\ldots ,X^{\left(k\right)}\right\}$ obtained from the initial PDFs π(X), the optimum exterior camera orientation values are calculated (this is explained in more detail in Section 3.2). Then, the estimated image points $M(X^{\left(l\right)})$, for l=1,…,k, are calculated with Equation (6) and compared with their corresponding pixel $m_{i}$ using the likelihood Equation (13). This gives *k* likelihood values $L^{\left(l\right)}\left(m\left|X^{\left(l\right)},\sigma^{2(l)}\right.\right)$, which quantify the quality of the image points prediction produced by the interior camera orientation set $X^{\left(l\right)}$. The best set of values in sample $\left\{X^{\left(1\right)},\ldots ,X^{\left(k\right)}\right\}$ is the one that maximizes the likelihood function (Equation (13)). From a metrological point of view and according to Bayes’ theorem, this is the best set of interior camera orientations for the sample considered but not the only solution. Therefore, several groups of samples of the interior camera orientation have to be generated, and in each group, the best set of values has to be selected according to its likelihood score. In this way, a distribution of the possible values of X is constructed with Equation (12), considering the real points of the image and the prior distributions π(X) and $\pi (\sigma^{2})$.

Finally, we can write the new form of the posterior distribution from Equation (12) by introducing the likelihood from Equation (13):

(14) $$\pi \left(X,\sigma^{2}\left|m\right.\right)=\frac{1}{Z}\pi \left(X\right)\pi \left(\sigma^{2}\right)\prod_{i=1}^{N}\frac{1}{\sqrt{2\pi \sigma^{2}}}\times exp\left(-\frac{1}{2\sigma^{2}}{\left(m_{i}-M\left(X\right)\right)}^{T}\left(m_{i}-M\left(X\right)\right)\right)$$

In practice, the posterior distribution of Equation (14) does not have a closed-form solution. This is mainly due to the integration that must be solved to obtain the model evidence or marginal likelihood *Z*, which can be difficult or impossible to calculate. For this reason, numerical methods based on Markov Chain Monte Carlo (MCMC) are often used to calculate $\pi \left(X,\sigma^{2}\left|m\right.\right)$ [46]. With the MCMC, the posterior distribution is proportional to the prior distribution times the likelihood [48,49], i.e., $\pi \left(X,\sigma^{2}\left|m\right.\right)\propto \pi \left(X\right)\pi \left(\sigma^{2}\right)L\left(m\left|X,\sigma^{2}\right.\right)$.

### 3.1. Markov Chain Monte Carlo (MCMC)

The MCMC method uses Markov chains to perform Monte Carlo estimation. The Markov chain represent a sequence of events where the probability of the future $x_{t+1}$ only depends of the present $x_{t}$, i.e., it is memoryless (Markov Property). MCMC allows us to obtain a sequence of random samples of an (unknown) probability distribution from which direct sampling is not possible [48]. Therefore, the MCMC establishes a relationship between the a priori distributions and the a posteriori distributions. The initial values of the MCMC will not exactly follow the distribution of the posterior values—this phase is known as the burn-in period—but eventually, the predictions will tend towards the desired distribution (stationary state) [50]. The initial values are needed to move up the chain but are discarded when it is finished, i.e., they are not included in the final distribution. The MCMC converges to an invariant distribution after a number of steps; therefore, it is useful to obtain the a posteriori distribution [47].

The most common MCMC algorithms include Metropolis–Hastings, Adaptive Metropolis, and Hamiltonian Monte Carlo [47]. In this paper, the Adaptive Metropolis algorithm is used, as it has proven to be quite efficient in practice, allowing fast convergence in situations where other MCMC algorithms are impractical [51]. For instance, the Metropolis–Hastings algorithm needs to choose a proposed distribution that is as close as possible to the posterior distribution. This is not always possible in practical applications [52]. On the other hand, the Hamiltonian Monte Carlo algorithm is slower per iteration than most other approaches because it needs to compute the gradient at different points in the posterior distribution [47]. In addition, it is not widely used in practice because of the difficulty of adjusting the hyperparameters [53].

### 3.2. Computational Tools

Bayesian calibration produces a finite number of possible values rather than an optimal one. Therefore, it is necessary to fit it to an optimal PDF by Bayesian inference. In this work, both Bayesian calibration and PDF fitting were performed using the general-purpose uncertainty quantification framework UQLAB [47]. UQLAB is a reliable framework for Bayesian inversion and uncertainty quantification [46]. To perform the Bayesian calibration in UQLAB, it is essential to specify:

- The uncertain model parameters; in our case, the intrinsic parameters and lens distortion coefficients (interior orientation) $X=\left(A,k_{1},k_{2},k_{3},p_{1},p_{2}\right)$.
- The measurement vector or experimental data m.
- The forward model, i.e., M(X).

In our Bayesian inversion process, only the PDFs of the intrinsic parameters and lens distortion are updated. However, the calculation of the extrinsic parameters is necessary because the model M(X) is also dependent on them. Therefore, for each combination of intrinsic parameters and lens distortion sampled in the Bayesian process, $X^{\left(l\right)}=\left(A^{\left(l\right)},k_{1}^{\left(l\right)},k_{2}^{\left(l\right)},k_{3}^{\left(l\right)},p_{1}^{\left(l\right)},p_{2}^{\left(l\right)}\right)$, the proposed methodology estimates (R,t) for each image from its corresponding homography $H\;=\;\left[h_{1},h_{2},h_{3}\right]$. According to Zhang [6], this can be implemented as follows:

(15) $$\begin{array}{cc} r_{1} & =\lambda A^{-1}h_{1}\\ r_{2} & =\lambda A^{-1}h_{2}\\ r_{3} & =r_{1}\times r_{2}\\ t & =\lambda A^{-1}h_{3} \end{array}$$

with a scale factor:

$$\begin{array}{c} \lambda =\frac{1}{∥A^{-1}h_{1}∥}=\frac{1}{∥A^{-1}h_{2}∥} \end{array}$$

Homography H is computed with the ideal image points, i.e., after correcting lens distortions (with numeric nonlinear least-squares optimization) in each Bayesian inverse model simulation.

According to [6], the matrix $R=\left[r_{1},r_{2},r_{3}\right]$ computed with Equation (15) may not be a true rotation matrix because of the noise in the data. It is, therefore, recommended to compute the best or true rotation matrix by singular value decomposition (SVD). However, we believe that both the best estimates of R-matrix and t-matrix may not be the most optimal due to non-linearity. Therefore, we use the R and t values from the above expressions (for each $X^{\left(l\right)}$) as initial guests of a nonlinear model to be optimized:

(16) $$\sum_{i=1}^{n}\sum_{j=1}^{m}{∥m_{i,j}-M\left(R_{i}^{\left(l\right)},t_{i}^{\left(l\right)}\right)∥}^{2}$$

where $M(R_{i}^{\left(l\right)},t_{i}^{\left(l\right)})$ is the estimated image point computed by Equation (6), corresponding to the set $X^{\left(l\right)}$. Therefore, for each set $X^{\left(l\right)}$, there exists an optimal solution of $R^{\left(l\right)}$ and $t^{\left(l\right)}$ that minimizes Equation (16).

This optimization was performed using the Levenberg–Marquardt algorithm implemented in Matlab [33]. Once $R^{\left(l\right)}$ and $t^{\left(l\right)}$ are known, the Bayesian inference-based method searches for the possible values of the interior camera orientation that minimize Equation (10).

The steps to apply the proposed Bayesian inference-based method for the uncertainties characterization are:

1. Calculate the intrinsic parameters and distortion coefficients with their uncertainties using Zhang´s method [6] (with Matlab or OpenCv), assuming normal distributions.
2. With each set of random values sampled from the PDFs of the intrinsic parameters of the camera and lens distortion coefficients, obtain the undistorted image points.
3. With the undistorted points and the sampled values, calculate the exterior camera orientation or extrinsic parameters with Equation (15).
4. Use the obtained exterior camera orientation values as initial guests and determine the optimal value of the exterior camera orientation using Equation (16).
5. Calculate the theoretical image points with Equation (6).

**Figure 2:**
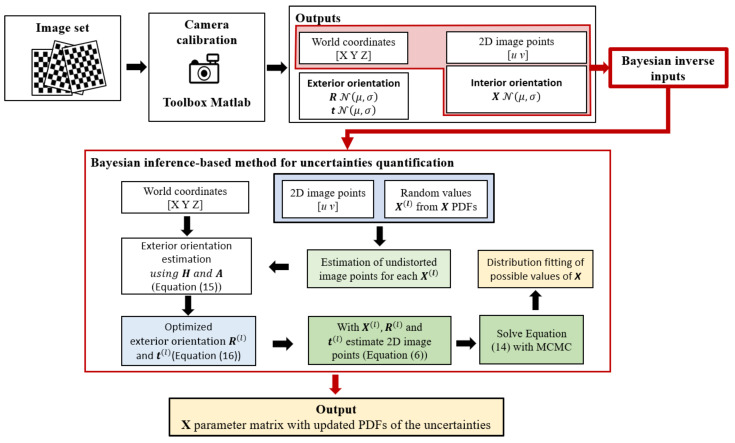
Bayesian inference-based method for uncertainties quantification of the interior camera orientation.

Figure 2 shows a schematic representation of the Bayesian inference-based method for the uncertainties characterization of the interior camera orientation.

## 4. Results

Table 1, Table 2 and Table 3 show the interior camera orientation results corresponding to three image sets obtained using the Matlab camera calibration software and then Bayesian calibration. These sets of images were chosen because they are the most interesting of the five sets analyzed. For the Bayesian method, 5000 steps and 50 Markov chains were used, so a total of 25,000 simulations per image set were performed, with a computational cost of approximately 5 h. It was found that in most cases, the differences between the mean values of the interior camera orientation obtained with the two methods are not significant (less than 5%). However, the differences between the values of the standard deviations are relevant and depend on the set of images considered. It can also be seen that the normality assumption of the method commonly used to estimate the uncertainties of Zhang’s method is not always met. In this case, the appropriate PDFs are those obtained using the proposed Bayesian inference-based method, as this is a mathematical approach specifically designed to generate a probability distribution for uncertain parameters from prior knowledge [54,55,56].

To verify the effects of the different PDFs found in Table 1, Table 2 and Table 3, it is necessary to perform a statistical comparison of both the estimated points and the re-projection error. Therefore, in this work, we have verified the effect of the uncertainties of the camera parameters through a propagation analysis and uncertainty quantification. Therefore, we have calculated the estimated image points with Equation (6), using three different procedures:

1. **Procedure A**: Based on 1000 pseudo-random samples from the Gaussian PDFs of the interior and exterior camera orientations, computed with the Matlab camera software. In this way, we have then assumed that the exterior camera orientation do not depend on the other parameters of the camera.
2. **Procedure B**: From 1000 pseudo-random samples, considering only the Gaussian PDFs of the interior camera orientation calculated with the Matlab camera software. This is the common procedure used for an already calibrated camera [6], i.e., with the X-values provided by the Matlab camera software, executing the following steps:
  - First, the exterior camera orientation matrices [R,t] are calculated with Equation (15).
  - Then, the rotation matrix R is optimized using SVD.
  - Finally, the image points are predicted with Equation (6).
3. **Procedure C**: From 1000 pseudo-random samples from the PDFs of the interior camera orientation obtained with the proposed Bayesian inference method, where, in addition to each sample of the interior camera orientation, the optimal values of R and t are obtained. In this case, the procedure used to calculate the estimated point was as follows:
  - First, the initial exterior camera orientation matrices (R,t) are calculated using Equation (15) with the random values from the X PDFs.
  - Then, R and t are optimized with the Equation (16), as was carried out in the proposed Bayesian camera calibration procedure.
  - Finally, the image points are predicted with Equation (6).

**Figure 3:**
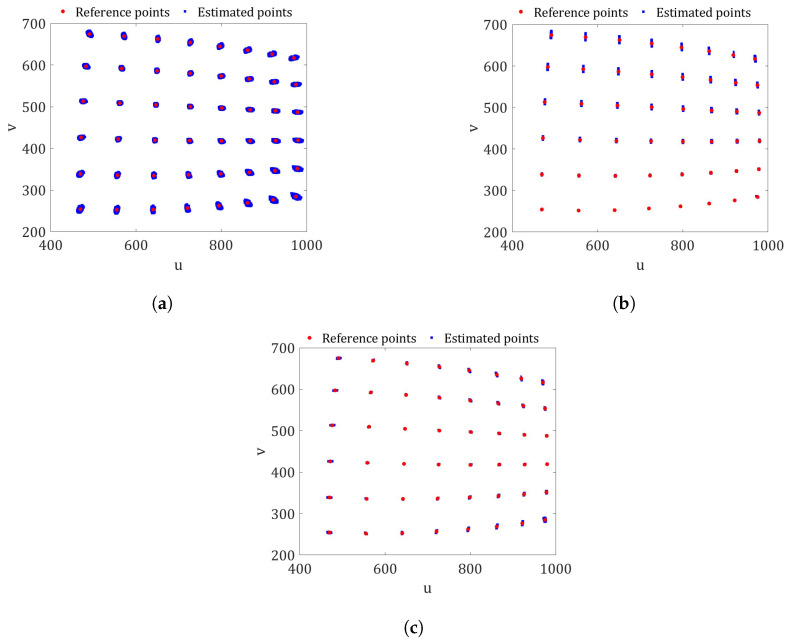
The comparison between the estimates and pixel coordinates for the checkerboard in Figure 1a. (**a**) Procedure A: PDFs of both X and (R,t) estimated using the Matlab toolbox. (**b**) Procedure B: PDFs of X and the possible values of (R,t) using Equation (15). (**c**) Procedure C: PDFs of X and the possible values of (R,t) optimized with Equation (16) (proposed method).

Figure 3a shows the result of the comparison between the reference image points (red color) and the estimated ones (blue color) obtained with Procedure A. The reference points are the coordinates of the corners of the image checkerboard shown in Figure 1a and represent our ground truth. The reference points were detected using the Matlab command *detectCheckerboardPoints* [33]. This command is based on the work of Geiger et al. [9]. It can be seen that the reference points are within the distribution of the estimated checkerboard corners. However, their variability is much higher compared to Figure 3b,c, corresponding to Procedures B and C, respectively. The higher variability is due to the non-linearity of the complete camera model. Therefore, the uncertainties of the exterior camera orientation resulting from the Matlab camera software should not be used in an uncertainty propagation analysis.

From a validation point of view, no major differences are observed between the distributions of the estimated image points in Figure 3b and those in Figure 3c. Therefore, it could be said that, in this case, the estimation of the exterior camera orientation using the actual method (Equation (15)) is similar to the proposed optimal solution obtained from Equation (16).

**Figure 4:**
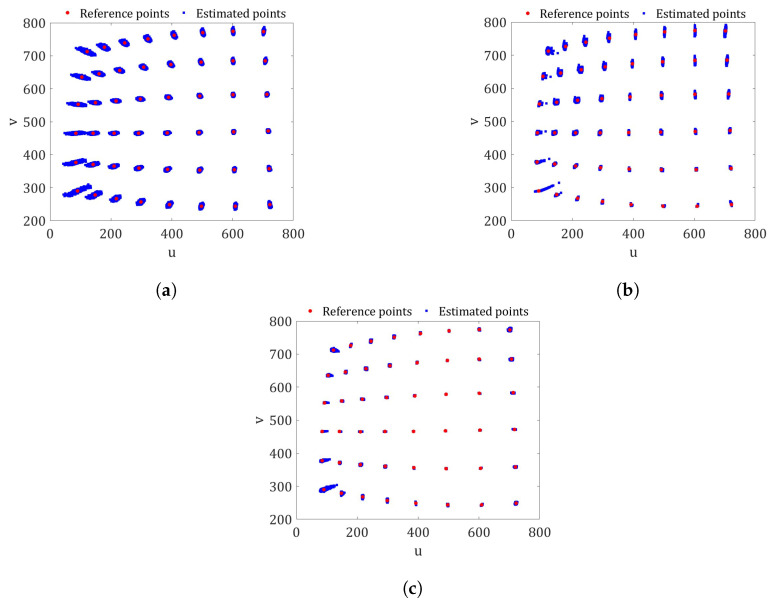
The comparison between the estimates and pixel coordinates for the checkerboard in Figure 1e. (**a**) Procedure A: PDFs of both X and (R,t), estimated using the Matlab toolbox. (**b**) Procedure B: PDFs of X and the possible values of (R,t) using Equation (15). (**c**) Procedure C: PDFs of X and the possible values of (R,t), optimized with Equation (16) (proposed method).

However, if the same three different uncertainty quantification procedures are carried out with a different set of images, the differences in the quality of the prediction points of the checkerboard image arise when comparing the results of the procedures. Figure 4 shows the distributions of the possible values of the image checkerboard corners (Estimated points) and pixel coordinates (Reference points) shown in the Figure 1e belonging to set 3 for the three procedures. The estimated points were calculated using the camera parameter values from Table 2. It can be seen that in the three cases shown, the estimated points closest to the vertical axis *v* have a greater distortion than the rest of the estimated points due to the barrel distortion. However, this unfavorable effect is minimized when the predictions of the image points are made with the PDFs from the Bayesian calibration and the exterior camera orientations are calculated with Equation (16) (Procedure C), as is depicted in Figure 4c.

**Figure 5:**
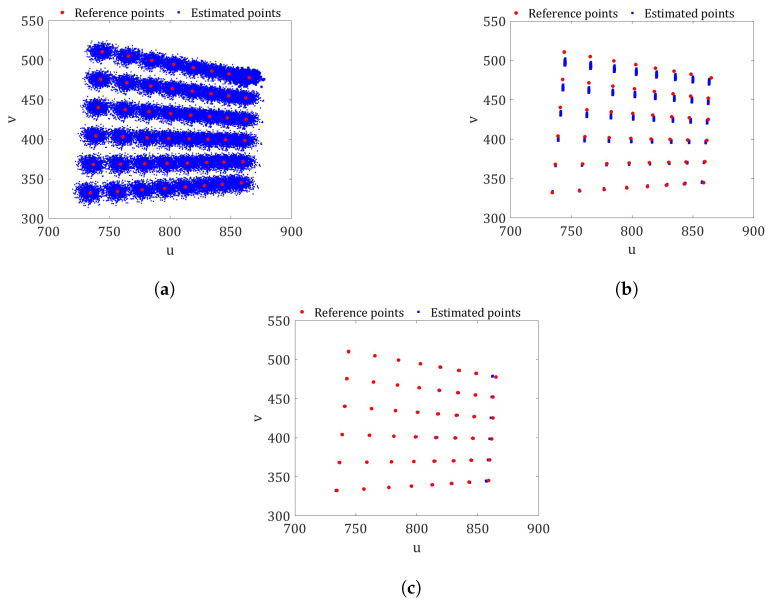
The comparison between the estimates and pixel coordinates for the checkerboard in Figure 1j. (**a**) Procedure A: PDFs of both X and (R,t) estimated using the Matlab toolbox. (**b**) Procedure B: PDFs of X and the possible values of (R,t) using Equation (15). (**c**) Procedure C: PDFs of X and the possible values of (R,t) optimized with Equation (16) (proposed method).

The worst results were obtained with the checkerboard shown in Figure 1j. Figure 5 shows the distribution of the possible checkerboard corner values (estimated points) and pixel coordinates (reference points). The greatest dispersion appears when using the uncertainty propagation of Procedure A (Figure 5a). In this case, the reference image points lie within the estimated points. However, the error is very large because the distributions of the estimated points overlap. Figure 5b shows that in most cases, the predictions made using the Matlab Gaussian PDFs of the interior camera orientation and the values of (R,t) calculated using Equation (15) (Procedure B) do not match the reference points. In addition, the distributions of the estimated point values differ significantly from the ones in Figure 5a. This is because the effect of non-linearity is much greater in this image than in the rest of the images analyzed. As already observed in the cases analyzed previously (Figure 3c and Figure 4c), when the PDFs of the Bayesian method are used and (R,t) are optimized with Equation (16), i.e., when applying the uncertainty propagation of Procedure C, both the variance and the quality of the prediction are improved. However, in some areas, the reference points are not within the distribution of the possible estimated points.

**Figure 6:**
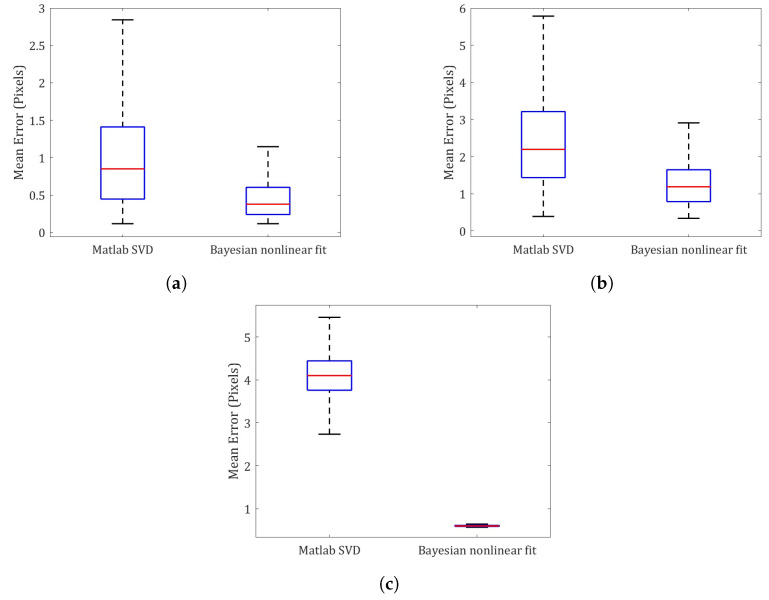
Re-projection error statistical comparison between Procedure B (Matlab SVD ) and Procedure C (Bayesian nonlinear fit). (**a**) Checkerboard of Figure 1a. (**b**) Checkerboard of Figure 1e. (**c**) Checkerboard of Figure 1j.

Figure 6 shows the statistical re-projection errors (average Euclidean norm) between the estimated points and the reference points for the checkerboards in Figure 1a,e,j. In this case, the re-projection errors calculated with the PDFs of both X and (R,t) from the Matlab camera software (Procedure A), are not shown because their variance is very high, i.e., those corresponding to the estimates in Figure 3a, Figure 4a, Figure 5a. The whisker bounds represent a 95% confidence interval. In both Figure 6a,b, we observe an asymmetric distribution of the errors of both Matlab based on SVD and the proposed Bayesian method (optimization of the exterior camera orientation). In both cases, the asymmetry tends toward the minimum values and is more pronounced when the PDFs from the Bayesian calibration are used. The largest difference between the re-projection errors of the two methods is depicted in Figure 6c, which corresponds to the estimates of the points of the checkerboard in Figure 1j. The distribution of the possible values of the re-projection error shows that the accuracy obtained from the proposed Bayesian calibration PDFs is significantly higher than the one obtained with the Matlab camera software (based on SVD).

To explain the differences in the statistical re-projection errors between the two calibration methods, the Matlab re-projection errors were recalculated, but this time, the exterior camera orientation matrices were optimized as the Bayesian calibration process proposed. We call this case “Matlab nonlinear fit”.

**Figure 7:**
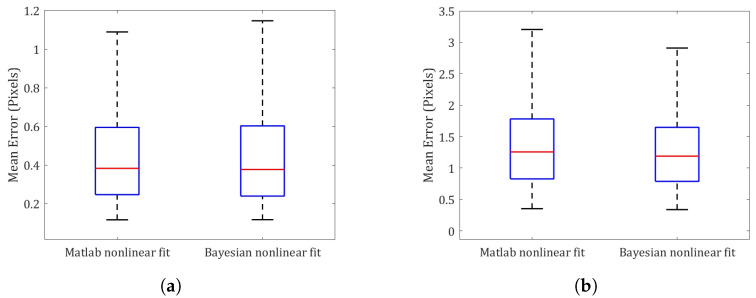

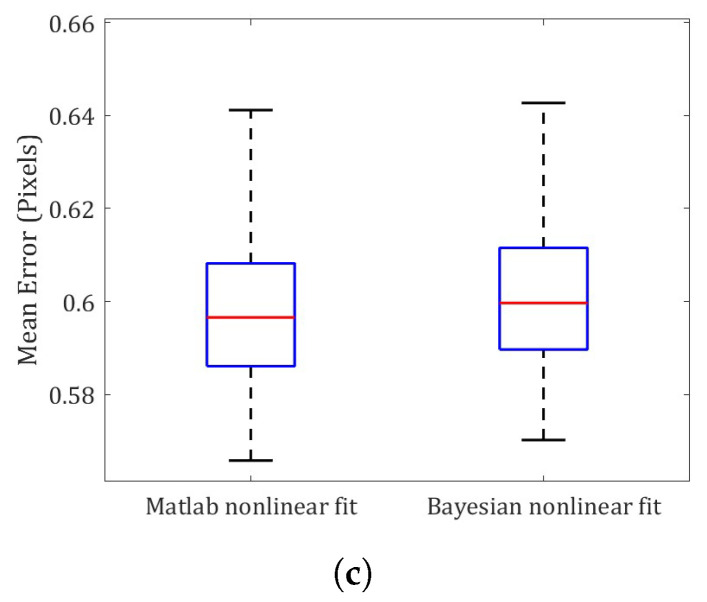
Re-projection error statistical comparison between Matlab nonlinear fit and Procedure C (Bayesian nonlinear fit). (**a**) Checkerboard of Figure 1a. (**b**) Checkerboard of Figure 1e. (**c**) Checkerboard of Figure 1j.

Figure 7 shows the new re-projection errors estimated with the Matlab nonlinear fit versus the proposed Bayesian calibration. It can be seen that the predictions made with the interior camera orientation PDFs from the Matlab camera software are significantly improved by the optimization of the exterior camera orientation. Thus, for the set of images analyzed, the difference between the Matlab X PDFs and the Bayesian calibration does not significantly affect the predictions of the image points but only when calculating the exterior camera orientation, as proposed in our Bayesian inference-based method.

## 5. Discussion

Computer vision has a wide range of applications in today’s world. To ensure its reliability, it is, therefore, necessary that all the measurements made with the different algorithms used are presented with their respective uncertainty to quantify their accuracy.

Images are one of the most commonly used data for computer vision; that is why cameras have become an essential part of this technique. They capture points in the 3D world and project them onto a 2D plane, which we see as images. To extract accurate information from the captured images, a metrological characterization of the intrinsic and distortion lens parameters of the cameras used is, thus, essential.

The high dissemination of made-up camera calibration algorithms in the web, implemented in software such as Matlab or OpenCV, can be extremely helpful as they provide powerful solutions for non-expert users. However, these algorithms must be “handled with care”, because the uncertainty quantification in the camera calibration process demands a deep knowledge on metrology issues.

The methodology proposed by Zhang [6] is extensively used in camera calibration software such as those mentioned before. However, no study has yet been carried out to validate the uncertainties of interior camera orientation calculated with this type of tool. Therefore, we have used the Bayesian inversion to verify the PDF uncertainties for both the intrinsic camera parameters and lens distortion coefficients of the Matlab software. This approach does not replace the calibration method proposed by Zhang [6]. It verifies the practical use of the uncertainties calculated with the considered camera software.

On a first instance, it is worth commenting that the PDFs of the interior camera orientation found with the Bayesian inference are not always normal as the ones assumed by the Matlab software. Moreover, we have demonstrated that in an uncertainty propagation study, the PDFs of the interior camera orientation cannot be used directly in conjunction with the exterior orientation provided by Matlab, as the model is non-linear. Thus, the linear simplification of the software, results in a very large scatter of possible pixel values and a high statistical re-projection error. The assumptions of Matlab for the calculation of camera parameter uncertainties are conditioned by the image pose considered in the calibration. Therefore, to quantify the quality of the camera calibration, it is necessary to verify the effect of camera parameter uncertainties on the estimated image points, as was performed in this research. This is an important finding since, as in transport and industry, the decision-making activities rely more and more on computer vision, and it is compulsory to determine the metric reliability of the images. The uncertainty quantification becomes then an unavoidable issue.

We have also developed the uncertainty propagation of the interior orientation of the camera from Matlab in accordance with the procedure implemented in this software. This method uses the homography matrix and the SVD to compute the true values of the rotation matrix. We have found that the accuracy of the point estimates with this procedure is also strongly affected by the image considered. Therefore, we propose to always optimize the extrinsic parameters or exterior camera orientation with Equation (16), using the Matlab [R,t] matrices as initial values. In this way, the predictions of the image points are significantly improved and are similar to the results obtained with the PDFs of the Bayesian calibration process.

However, we are aware that these findings were obtained with a reduced number of images and that to ensure a good accuracy in the magnitudes of the camera parameters, it was proven [28,29,30] that it is necessary to perform an analysis with a larger number of images. As the Bayesian inference is a very time-consuming procedure, we propose, as a future work, the development of surrogate models to efficiently perform a reliable uncertainty quantification.

## 6. Conclusions

It is a fact that uncertainty quantification in camera calibration is currently an unavoidable issue. This work presents the verification of the uncertainties of the interior camera orientation calculated with the Matlab camera software. Therefore, we have analyzed the impact of the assumptions made by the software to quantify the uncertainties of Zhang´s camera calibration method.

We have found that the PDFs of the Bayesian inverse process differ from those assumed by the Matlab camera calibration software. We have also shown that using the PDFs obtained in Matlab can lead to poor estimates of the image points, since the assumptions on which the software relies to estimate the uncertainty of the camera parameters are strongly conditioned by the checkerboard pose used.

Furthermore, we have demonstrated that the main source of bias in the prediction of image points is the method used to calculate the camera’s exterior orientation. According to the results obtained in the present work, if the exterior orientation of the camera is optimized with the procedure we have proposed, the impact of the Gaussian assumption of the interior camera orientation in the statistical re-projection errors is minimized. We, therefore, recommend optimizing the camera’s exterior orientation based on the interior orientation values, as this will significantly improve the accuracy of the image point predictions.

Moreover, a larger number of images should be considered to improve the accuracy of the camera parameters, and factors such as image light and time drift of the camera parameters have to be computed for a more realistic uncertainty quantification of a vision system.

The novel Bayesian inference-based approach for the uncertainty characterization of Zhang’s camera calibration method may require a higher computational cost compared with the uncertainty analysis of conventional camera calibration software.

In this sense, we propose, as a next step, to conjugate the Bayesian inference with surrogate models. This will allow us to fulfill two opposite but necessary requirements: to reduce the computational cost of uncertainty quantification and to ensure the metrological reliability of the results.

## Figures and Tables

**Table 1:** Statistical moments and PDFs for image subset 1.

	Zhang’s Method			Proposed Bayesian Method		
Parameter	Mean	Standard Deviation	PDF	Mean	Standard Deviation	PDF
$f_{x}$	565.0326	3.3316	Gaussian	564.9714	3.1788	Logistic
$f_{y}$	565.5575	3.3106	Gaussian	565.3179	3.5436	Weibull
$c_{x}$	647.1847	0.6420	Gaussian	647.1668	0.8688	Gaussian
$c_{y}$	504.2582	0.6714	Gaussian	504.3673	1.0146	Gaussian
$k_{1}$	−0.2693	0.0037	Gaussian	−0.2700	0.0040	Gumbel
$k_{2}$	0.1244	0.0068	Gaussian	0.1252	0.0071	GumbelMin
$k_{3}$	−0.0467	0.0063	Gaussian	−0.0458	0.0062	Gaussian
$p_{1}$	−0.0022	0.0003	Gaussian	−0.0022	0.0005	Gaussian
$p_{2}$	0.0026	0.0002	Gaussian	0.0027	0.0003	Beta

**Table 2:** Statistical moments and PDFs for image subset 3.

	Zhang’s Method			Proposed Bayesian Method		
Parameter	Mean	Standard Deviation	PDF	Mean	Standard Deviation	PDF
$f_{x}$	566.8778	3.6615	Gaussian	567.4637	3.5116	Gaussian
$f_{y}$	567.4131	3.7248	Gaussian	567.9525	3.6931	Gaussian
$c_{x}$	651.4962	0.6736	Gaussian	651.7141	0.9339	Uniform
$c_{y}$	502.6545	0.7893	Gaussian	502.7747	1.0737	Uniform
$k_{1}$	−0.2465	0.0036	Gaussian	−0.2466	0.0029	Gaussian
$k_{2}$	0.0756	0.0023	Gaussian	0.0756	0.0021	Gaussian
$k_{3}$	−0.0116	0.0006	Gaussian	−0.0117	0.0006	Gaussian
$p_{1}$	−0.0010	0.0002	Gaussian	−0.0010	0.0003	Gaussian
$p_{2}$	−0.0002	0.0002	Gaussian	−0.0002	0.0003	Gaussian

**Table 3:** Statistical moments and PDFs for image subset 5.

	Zhang’s Method			Proposed Bayesian Method		
Parameter	Mean	Standard Deviation	PDF	Mean	Standard Deviation	PDF
$f_{x}$	553.2964	4.5922	Gaussian	552.7039	3.3532	Gaussian
$f_{y}$	548.7821	4.3422	Gaussian	547.2856	3.6085	Uniform
$c_{x}$	641.1869	2.1851	Gaussian	641.2931	2.9777	Gumbel
$c_{y}$	502.4452	2.2514	Gaussian	502.2640	2.8074	Weibull
$k_{1}$	−0.2400	0.0051	Gaussian	−0.2396	0.0037	Logistic
$k_{2}$	0.0766	0.0047	Gaussian	0.0764	0.0038	Logistic
$k_{3}$	−0.0140	0.0015	Gaussian	−0.0141	0.0013	Logistic
$p_{1}$	−0.0012	0.0006	Gaussian	−0.0011	0.0006	Gaussian
$p_{2}$	0.0035	0.0005	Gaussian	0.0036	0.0005	Beta

## Data Availability

Not applicable.
